# Impact of ultrasound on management for dyspnea presentations in a Rwandan emergency department

**DOI:** 10.1186/s13089-019-0133-8

**Published:** 2019-08-28

**Authors:** Olivier Felix Umuhire, Michael B. Henry, Adam Carl Levine, Giles N. Cattermole, Patricia Henwood

**Affiliations:** 1 Department of Anesthesia, Critical Care and Emergency Medicine, University of Rwanda, Kigali, Rwanda; 2 Columbia University Vagelos College of Physicians and Surgeons, New York, USA; 3 The Warren Alpert Medical School of Brown University, Providence, RI USA; 4 Department of Emergency Medicine, King’s College/NHS, London, UK; 5 Department of Emergency Medicine, Brigham and Women’s Hospital, Boston, USA

**Keywords:** Ultrasound, Dyspnea, Rwanda

## Abstract

**Background:**

The complexity of diagnosis for critically ill dyspnea presentations in the emergency department remains a challenge. Accurate and rapid recognition of associated life-threatening conditions is paramount for timely treatment. Point-of-care ultrasound (POCUS) has been shown to impact the diagnosis of dyspnea presentations in resource-rich settings, and may be of greater diagnostic benefit in resource-limited settings.

**Methods:**

We prospectively enrolled a convenience sample of 100 patients presenting with dyspnea in the Emergency Department at University Teaching Hospital of Kigali (UTH-K) in Rwanda. After a traditional history and physical exam, the primary treating team listed their 3 main diagnoses and ranked their confidence accuracy in the leading diagnosis on a Likert scale (1–5). Multi-organ ultrasound scans performed by a separate physician sonographer assessed the heart, lungs, inferior vena cava, and evaluated for lower extremity deep vein thrombosis or features of disseminated tuberculosis. The sonographer reviewed the findings with the treating team, who then listed 3 diagnoses post-ultrasound and ranked their confidence accuracy in the leading diagnosis on a Likert scale (1–5). The hospital diagnosis at discharge was used as the standard in determining the accuracy of the pre- and post-ultrasound diagnoses.

**Results:**

Of the 99 patients included in analysis, 57.6% (*n* = 57) were male, with a mean age of 45 years. Most of them had high-level acuity (54.5%), the dyspnea was of acute onset (45.5%) and they came from district hospitals (50.5%). The most frequent discharge diagnoses were acute decompensated heart failure (ADHF) (26.3%) and pneumonia (21.2%). Ultrasound changed the leading diagnosis in 66% of cases. The diagnostic accuracy for ADHF increased from 53.8 to 100% (*p* = 0.0004), from 38 to 85.7% for pneumonia (*p* = 0.0015), from 14.2 to 85.7% for extrapulmonary tuberculosis (*p* = 0.0075), respectively, pre and post-ultrasound. The overall physician diagnostic accuracy increased from 34.7 to 88.8% pre and post- ultrasound. The clinician confidence in the leading diagnosis changed from a mean of 3.5 to a mean of 4.7 (Likert scale 0–5) (*p* < 0.001).

**Conclusions:**

In dyspneic patients presenting to this Emergency Department, ultrasound frequently changed the leading diagnosis, significantly increased clinicians’ confidence in the leading diagnoses, and improved diagnostic accuracy.

## Background

Dyspnea, or shortness of breath, is one of the most common complaints in the emergency department, accounting for over three million visits yearly in the United States [1]. This medical condition requires urgent and accurate assessment by the medical practitioner in the emergency department (ED) to provide appropriate initial therapy that can improve clinical outcomes. The broad differential diagnosis for dyspnea makes it challenging to initially manage, particularly in limited-resource settings.

The surge of cardiovascular disease in Africa, combined with longstanding infectious diseases, such as human immunodeficiency virus (HIV) and tuberculosis (TB), increases the likelihood of patients presenting with acute dyspnea, especially if the underlying condition is undiagnosed or the patient is not compliant with primary care therapies [2, 3].

There is an extensive cardiopulmonary differential diagnosis for dyspnea (e.g. pulmonary embolism (PE), pneumonia, acute decompensated heart failure (ADHF), pneumothorax) with potential for evaluation by ultrasound. Even for the life-threatening conditions listed above, it is clear that there are many challenges for emergency care providers in the management of the dyspneic patient. Several studies have shown that the traditional pathway of history, physical exam and chest X-ray is not always accurate in making the diagnosis [4–6]. Moreover, resource-limited settings are challenged by the lack of readily available and accessible diagnostic tools.

Ultrasound is becoming a more widespread tool as it is non-invasive, without ionizing radiation, cost-effective, and rapid if performed by trained clinicians at the bedside [7]. Ultrasound, particularly that at the point-of-care (POCUS) is now emerging as standard practice in emergency medicine and critical care in North America and Europe, where it has been studied and described to help in diagnosing and treating several medical conditions [8–10]. The “triple scan” technique (focused heart, lung and inferior vena cava (IVC)) ultrasound evaluation has been shown to help make the diagnosis for dyspnea [10]. Several studies have proved ultrasound to be an essential tool in providing clinical information for guiding medical decisions in critically ill patients [11, 12]. Point-of-care ultrasound has shown significant diagnostic accuracy in an African context, especially when differentiating cardiac from pulmonary diseases [13].

This study aimed at incorporating ultrasound techniques as an adjunct to the history-taking and physical exam to orient critical medical decisions for dyspneic patients presenting to the ED. The primary objective of the study was to determine the proportion of cases presenting with acute dyspnea in which ultrasound changes the clinician’s diagnosis for the patient. A secondary objective of this study was to determine if and how often multi-organ ultrasound exams improve the accuracy and confidence of emergency care providers’ diagnosis in patients presenting with dyspnea in the Emergency Department at University Teaching Hospital-Kigali (UTH-K).

## Methods

### Study design and setting

This is a prospective, observational study enrolling a convenience sample of adult participants presenting with dyspnea to an urban Rwandan emergency department. We enrolled participants presenting to the Emergency Department at University Teaching Hospital of Kigali (UTH-K) over a 1-year period from January 2017 to January 2018. Ethical approval was obtained from the UTH-K Ethical Committee and the Institutional Review Board (IRB) of the College of Medicine and Health Sciences/University of Rwanda and the Partners Healthcare IRB prior to study initiation. Written consent was obtained from participants prior to participation or next of kin if the patient was too sick to consent.

UTH-K, the largest tertiary care hospital in Rwanda, is 500-inpatient bed hospital, which provides speciality surgical and medical services, emergency and critical care, as well as diagnostic services (laboratory and imaging) to approximately 2 million people of Kigali and serves as the country’s main referral hospital. This emergency department receives 25,000 visits per year mostly acutely ill patients, including trauma as well as non-trauma patients. Patients at UTH-K are either directly brought in the ED from home, street, or transferred from peripheral hospitals in Rwanda. All adult patients pass through the ED and are received by either emergency and critical care residents or general practitioners. Imaging modalities including X-ray and computed tomography (CT) scans when available. The emergency department has access to ultrasound equipment immediately at the point-of-care, and ultrasound training is a formal curricular component and required competency of the emergency medicine residency-training program.

### Participants

The study included a convenience sample of participants triaged as Yellow, Orange or Red according to the Triage Early Warning Score/SAT-TEWS [14] who complained of breathlessness. Participants were adults, age > 16 years. All others were excluded. Patients were only enrolled when the principal investigator (PI) was present in the department; which was mainly during daytime hours any day of the week.

### Data collection

The PI, an emergency medicine physician with focused ultrasound training consistent with international recommendations [15] performed the ultrasound scans (including Heart–Lung-IVC “triple scan”, FASH and DVT studies) after history and physical exam by the treating medical team, but before other diagnostic studies were completed.

Ultrasonography features of pneumonia, pneumothorax, hemothorax, DVT, pulmonary embolism, ADHF, acute COPD or asthma exacerbation, pulmonary edema, pericardial effusion, pleural effusion and extra-pulmonary tuberculosis were recorded.

After initial evaluation by ED staff, general practitioners and residents/registrars, the PI asked the clinical team to generate their top 3 presumed diagnoses, and rank their confidence in the leading diagnosis on a Likert scale (1–5) after which ultrasound was then completed by the PI. The PI did not perform an independent physical exam but some findings were evident on visual inspection during the ultrasound (tachypnea, work of breathing, diaphoresis, abdominal distention, etc.), so the PI was not overall blinded to the clinical findings. Ultrasound findings were communicated to the treating team, after which the treating team reassessed post-ultrasound presumed diagnoses, ranked their confidence in the leading diagnosis. Follow-up was done to obtain diagnosis at discharge for the enrolled participants. The hospital diagnosis at discharge was used as the standard for comparison in determining the accuracy of the pre- and post-ultrasound diagnoses. The lead sonographer received dedicated ultrasound training over a 3-year period, was enrolled in an ultrasound fellowship and had passed an ultrasound observed structured clinical skills assessment prior to leading this study. Another ultrasound-fellowship trained investigator for quality assurance purposes reviewed random samples of 5% studies and there was no discordance with interpretation. A SonoSite M-Turbo® ultrasound machine (FUJIFILM SonoSite, Inc, Bothell, WA) was utilized to scan patients, and ultrasound clips were saved on a flash drive and then stored on an external hard disk. We used REDCap (Research Electronic Data Capture), a web-based electronic capture to enter and store the data [16].

### Statistical analysis

The primary objective of the study was to determine the proportion of cases presenting with acute dyspnea in which ultrasound findings change the clinician’s diagnosis for the patient. Based on some prior literature and our prior experience in this setting, we estimated this to be about 40% of cases. We wanted to ensure that the margin of error around our estimate is less than 10%. We used the standard formula for calculating the sample size needed to estimate a proportion in a large population of patients: *n* = (*z*_a/2_^2^* *p* * (1 − *p*))/*d*^2^). In our case, using the z distribution with an alpha of 0.05, the sample size needed was calculated to be 93 patients. We planned to oversample by about 5% to allow for missing follow up data in patients.

Descriptive statistics, including proportions for categorical data and means for normally distributed continuous data, were obtained using Microsoft Excel 2011 to identify characteristics of the dyspneic patients and the accuracy of physician diagnosis. Categorical variables were compared using Pearson Chi-square or Fisher’s exact test, as appropriate. Finally, student’s *t* test was used for comparing the means for normally distributed data when appropriate (significance was set at *p*-value < 0.05).

### Ultrasound protocols

#### Heart and IVC

For each patient enrolled, four cardiac views were obtained (parasternal long, parasternal short, subcostal and 4-chambers views). A visual estimate of the LV function was completed and classified as normal, decreased, or hyperdynamic. Pericardial effusion was assessed, and the quality (simple or complex) and the size of the effusion (qualitatively small or large) were noted. We defined sonographic tamponade whenever pericardial effusion was associated with RA collapse and/or RV collapse during diastole plus a plethoric IVC. The IVC was evaluated by visual estimate and recorded as “flat” and “volume responsive” when collapsing was visualized; it was recorded “plethoric” and “non-volume responsive” when it was visualized to be full without any variation during both cycles of respiration; it was reported to be “normal” when neither flat or plethoric [13].

#### Lungs

**Fig. 1:**
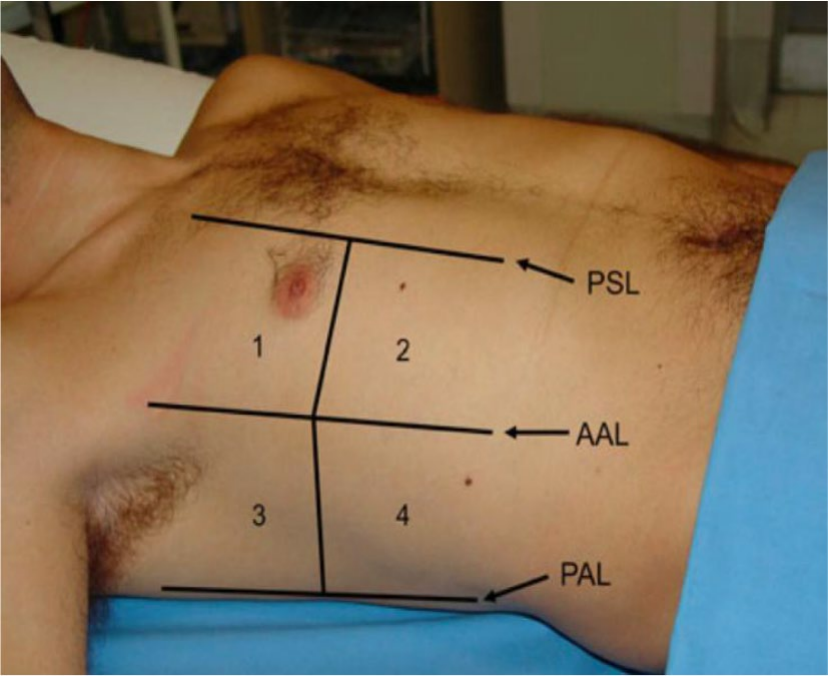
The areas of lung ultrasonography considered in the study. Areas 1 and 2: upper anterior and lower anterior; areas 3 and 4: upper lateral and basal lateral. Each area was the same on right and left side. AAL, anterior axillary line; PAL, posterior axillary line [11]

Lung ultrasound was performed following a 4-quadrant view protocol (Fig. 1) [11]. We started with 2 anterior views between vertical parasternal line (PSL) and the anterior axillary line (AAL). We then coupled with 2 views between the AAL and the posterior axillary line (PAL). The same protocol was applied to both the right and the left lung. Lung ultrasound was completed using a phased probe coupled with a high-frequency probe. The following features were recorded on both sides (right and left): lung sliding, pleural effusion small/large and simple/complex, B-lines focal/diffuse, subpleural/hepatization consolidation, and static/dynamic bronchograms.

#### Fash

**Fig. 2:**
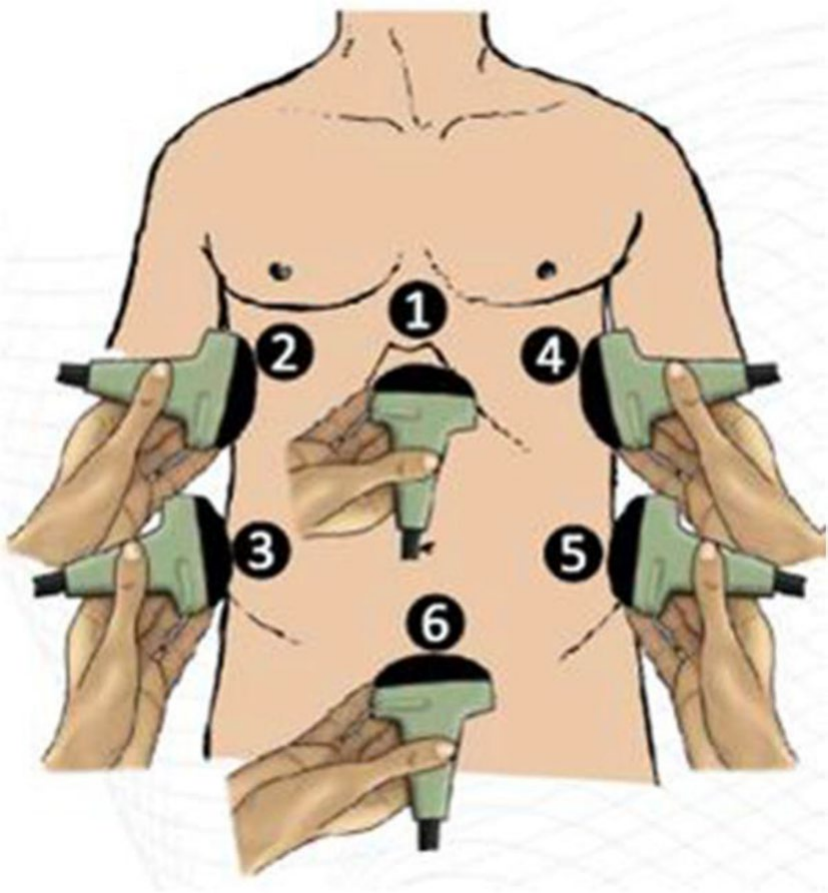
Schematic drawing of the ultrasound probe positions during the FASH examination [17]

Multi-organ ultrasonography included a FASH scan evaluating 6 zones (Fig. 2) which were described to have potential significant prediction for extra-pulmonary TB in HIV patients [17, 18]. FASH was incorporated into the scan protocol considering the local TB burden and clinical presentation of extra-pulmonary TB being very non-specific and difficult to diagnose, in addition to data on para-aortic nodes predicting pulmonary TB [19]. A low frequency curvilinear and a high-frequency linear probe were used for this application. Possible positive findings were: pleural effusion, pericardial effusion, hepatic and splenic microabscesses, ascites, and para-aortic lymphadenopathies.

#### DVT evaluation

A bedside ultrasound was also conducted to assess for lower extremity deep venous thrombosis. We used the two-point (two zone) DVT compression examination [20]. The region of common femoral vein (CFV) and the greater saphenous vein (GSV) bifurcation and the region of popliteal vein trifurcation were scanned for compressibility; a positive DVT scan was recorded whenever the vein was not compressible with enough pressure to deform the artery.

## Results

During the period of study 100 participants were enrolled. Among these, discharge data was missing for one (1%), and our analysis was conducted on the remaining 99. The study population was 57.6% (*n* = 57) male with a mean age of 45 years. Most participants enrolled were of high-level acuity per the TEWS triage category (54.5%)”red”. Many had dyspnea of acute onset (45.5%) as opposed to gradual or chronic and were transferred mainly from District Hospitals (50.5%) versus direct EMS or private transport. Table 1 describes the baseline population characteristics.

**Table 1:** Population characteristics (*n* = 9)

Age	45 ± 21.9 years
Gender	
Male	57 (57.6%)
Female	42 (42.4%)
Arriving from	
District Hospital (DH)	53 (53.5%)
Home	38 (38.4%)
Other	4 (4.0%)
Transport method	
DH ambulance	50 (50.5%)
Private	46 (46.5%)
EMS ambulance	3 (3.0%)
TEWS	
Red	54 (54.5%)
Orange	31 (31.3%)
Yellow	13 (13.1%)
Trauma	3 (3.0%)
Known HIV positive	17 (17.2%)
Dyspnea onset	
Acute (< 1 week)	45 (45.5%)
Gradual (1–4 weeks)	42 (42.4%)
Chronic (> 4 weeks)	12 (12.1%)

### Descriptive ultrasound findings

Decreased LV function was present in 28.3% of cases and hyperdynamic LV function in 33.3% of cases. The right lung ultrasound was reported to be abnormal in 80.8% of scans, whereas the left lung was reported to be abnormal in 70.7% of cases. Sonographic B-line pattern, pleural effusion, lung consolidation and air bronchograms were the most common sonographic features in abnormal lung ultrasounds among dyspneic patients. The volume responsiveness of patients was assessed by scanning the inferior vena cava (IVC); in 31.3% of cases patients were predicted volume responsive (flat IVC), 31.3% were normal and 37.4% were non-volume responsive (plethoric IVC). Positive deep venous thrombosis scans were infrequent (5.1%). Features of extrapulmonary tuberculosis were recorded 7% of the time, per the FASH protocol. Tables 2 and 3 describes the main sonographic findings.

**Table 2:** Cardiac and lung ultrasound findings (*n* = 99)

Cardiac exam	Normal	38 (38.4%)
	Any abnormality	61 (61.6%)
	Decreased LV function	28 (28.3%)
	Hyperdynamic LV	33 (33.3%)
	RV strain	8 (8.1%)
	Pericardial effusion	
	Present	25 (25.3%)
	Simple/complex	18 (18.2%)/7(7.1%)
	Small/large	21 (21.2%)/4 (4.0%)
	Tamponde	5 (5.1%)
Right lung exam	Normal	19 (19.2%)
	Any abnormality	80 (80.8%)
	Pleural effusion	47 (47.5%)
	Simple	30 (30.3%)
	Complex	17 (17.2%)
	Small	21 (21.2%)
	Large	26 (26.3%)
	B-lines	60 (60.6%)
	Focal	35 (35.4%)
	Diffuse	25 (25.3%)
	Consolidation	41 (41.4%)
	Air bronchograms	17 (17.2%)
	Non-sliding pleura	1 (1.0%)
Left lung exam	Normal	29 (29.3%)
	Any abnormality	70 (70.7%)
	Pleural effusion	39 (39.4%)
	Simple	24 (24.2%)
	Complex	15 (15.2%)
	Small	21 (21.2%)
	Large	18 (18.2%)
	B-lines	59 (59.6%)
	Focal	33 (33.3%)
	Diffuse	26 (26.3%)
	Consolidation	35 (35.4%)
	Air bronchograms	16 (16.2%)
	Non-sliding pleura	0 (0.0%)

**Table 3:** Other ultrasound findings (*n* = 99)

FASH exam	Normal	74 (74.7%)
	Any abnormality	25 (25.3%)
	Hepatic microabcesses	1 (1.0%)
	Splenic microabcesses	0 (0%)
	Ascites	22 (22.2%)
	Paraaortic LNs	2 (2.0%)
DVT exam	Normal	94 (94.9%)
	Non compressible	5 (5.1%)
IVC exam	Normal	31 (31.3%)
	Flat	31 (31.3%)
	Plethoric	37 (37.4%)

### Diagnostic accuracy

Multiorgan ultrasound was reported to change the clinician leading diagnosis in 65.7% of cases. The clinician confidence in the leading diagnosis changed from a mean of 3.5 to a mean of 4.7 (Likert scale 0–5) before and after ultrasound (*p* < 0.001).

The most common final diagnoses in this population were ADHF (26 patients, 26.3%) and pneumonia (21 patients, 21.2%). Clinicians correctly diagnosed ADHF in 14 patients (53.8%) pre-ultrasound compared to 26 patients (100%) post-ultrasound (*p* = 0.0004). The diagnosis of pneumonia was accurately made in 8 patients (38.0%) pre-ultrasound and in 18 patients (85.7%) post-ultrasound (*p* = 0.0015). Table 4 provides details of diagnostic changes pre- and post-ultrasound by final discharge diagnosis.

**Fig. 3:**
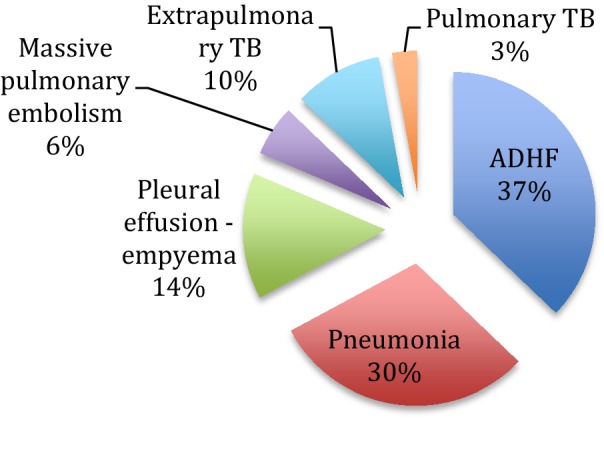
Hospital discharge diagnosis

Prior to ultrasound, the physician leading diagnosis matched the final discharge diagnosis in only 34.3% of cases; after ultrasound, the physician leading diagnosis matched the final discharge diagnosis in 89% of cases (Fig. 3).

**Table 4:** Pre- and post-POCUS diagnosis accuracy compared to discharge diagnosis

Discharge diagnosis	*N*	Pre-POCUS	Post-POCUS	*P* value
		Correct % (*n*)	Correct % (*n*)	
ADHF	26	53.8% (14)	100% (26)	*0.0004*
Pneumonia	21	38.0% (8)	85.7% (18)	*0.0015*
EPTB	7	14.2% (1)	85.7% (6)	*0.0075*
Massive PE	4	25.0% (1)	100% (4)	*0.0989*
Pleural effusion	10	10.0% (1)	100% (10)	*0.0002*
COPD	1	0% (0)	100% (1)	*0.7094*

## Discussion

This study was conducted in a low-resource setting emergency department, where patients presenting with acute dyspnea were assessed with multi-organ ultrasound, (evaluating the heart, lungs, IVC, and for features of disseminated TB or DVT) which often changed the leading clinical diagnosis, and improved the accuracy of the initial emergency clinician diagnosis relative to discharge diagnosis. In addition, physician confidence in their ED diagnosis significantly improved after ultrasound. Several critical medical conditions were recognized with a high accuracy relative to discharge diagnosis including ADHF (100%), pneumonia (85.7%), extrapulmonary TB (85.7%) and pleural effusion (100%).

Clinicians in resource-limited settings need to make timely, urgent management decisions for dyspneic patients, often with limited radiology capacity. This study demonstrates that incorporation of point-of-care ultrasound into the evaluation of dyspneic patients changed the leading ED diagnosis in most cases. Initiating urgent treatment to cover a broad differential diagnosis may be of limited benefit and can negatively impact patients and health care resource utilization in general. Narrowing the differential and changing the leading diagnosis with ultrasound may facilitate more appropriately tailored patient care.

This study reinforces conclusions from Mantuani et al. [10], who found POCUS to have 100% sensitivity for ADHF, and Nazerian et al. [8] who highlighted the usefulness of multi-organ POCUS when clinically predicting pulmonary embolism. Shah et al. [21] worked on the educational aspect of POCUS in low resource setting and proved its reproducibility in trained clinicians treating patients with dyspnea. A previous study in similar setting by Henwood et al. showed that POCUS had a significant impact on clinical decision-making [22]. Becker et al. [13] found that up to 14% of the clinician diagnostic accuracy changed after cardiopulmonary ultrasound in a trial done in Ghana among patients who presented with dyspnea. We suspect the higher percentage in our study may be due to the multi-organ scanning protocol and the wide range of diagnosis included.

This study is unique in that in addition to the studied “triple scan” of heart-lungs-IVC, and lower extremity DVT evaluation, we incorporated the FASH protocol [17]. This widened the range of use of ultrasound in patients with dyspnea extending its use from ADHF, pulmonary edema and COPD to infectious diseases such as pulmonary TB, extrapulmonary TB, and pneumonia. In this part of East Africa, where TB and HIV are endemic, incorporating ultrasound when managing HIV patients presenting with dyspnea could be of significant value. In addition, limited local availability of CT angiogram led to the addition of DVT assessment to further management of dyspneic patients with suspected pulmonary embolism.

Some aspects of our study deserve particular attention. ADHF accounted for 26.3% of the diagnoses, taking the lead among all the patients with acute dyspnea who presented in the ED. This highlights the continuing shift in the burden of acute disease from communicable to non-communicable diseases in Rwanda and sub-Saharan Africa in general [23]. Pneumonia could also be picked up more quickly and may be treated in a more timely fashion to fight sepsis, whose mortality is a global concern [23].

This study has several significant limitations. It was conducted at a single referral hospital in Rwanda, with a small sample size, enrolled at the convenience of a single sonographer investigator, most often during daytime hours. All of these factors could have impacted the included population and subsequent results. All ultrasound interpretations were also completed by the principal investigator, which could limit external validity. In addition, the leading diagnoses or clinical impressions of the treating teams may have varied from clinician to clinician based on their clinical judgment which is also a limitation.

A major limitation is that the available standard for comparison in our study was the hospital discharge diagnosis. While this was also utilized in previous similar work [10], its accuracy is limited by available diagnostic tests in this hospital. The results of the ultrasound itself may have influenced clinical diagnosis and final discharge diagnosis due to resource and logistical barriers in obtaining other gold standard testing in the hospital.

## Conclusion

Dyspnea remains a challenging clinical presentation in the emergency department, particularly in settings with limited portable or emergent diagnostic imaging modalities. Incorporating ultrasound findings with patient history and physical exam can change the initial emergency clinician diagnosis in a resource-limited setting and improve diagnostic accuracy. Further incorporation of point-of-care ultrasound into the routine management of dyspneic emergency department patients, particularly in settings with otherwise limited imaging resources, should be considered.

## Data Availability

All the scans obtained are available as well as data collection tools (corresponding author)

## Abbreviations

ACEP: American College of Emergency Physicians
ADHF: acute decompensated heart failure
ARDS: acute respiratory distress syndrome
CFV: common femoral vein
CMHS: College of Medicine and Health Sciences
COPD: chronic obstructive pulmonary diseases
CT: computed tomography
CXR: chest X-ray
DVT: deep venous thrombosis
ED: emergency department
FASH: focused assessment sonography in HIV and tuberculosis
GSV: greater saphenous vein
HIV: human immunodeficiency virus
IVC: inferior vena cava
Lt: left
LV: left ventricle
PE: pulmonary embolism
PI: primary investigator
POCUS: point-of-care ultrasound
RA: right atrium
REDCAP: research electronic data capture
Rt: right
TB: tuberculosis
TEWS: Triage Early Warning Score
UR: University of Rwanda
UTH-K: University Teaching Hospital of Kigali
